# Patient centered care in Islam: distinguishing between religious and sociocultural factors

**DOI:** 10.1186/2251-6581-12-30

**Published:** 2013-06-24

**Authors:** Asfandyar Khan Niazi, Sanjay Kalra

**Affiliations:** 1 Shifa College of Medicine, Pitras Bukhari Road, H-8/4, Islamabad, Pakistan; 2 Bharti Hospital & B.R.I.D.E., Karnal, India

**Keywords:** Patient centered care, Islam, South Asia, Diabetes

## Abstract

The use of patient centered care is promoted by Islam. Many of the countries with the highest percentage of diabetics are Muslim majority countries. The use of patient centered care in these areas is likely to reduce the burden of diabetes in these countries. However there are several challenges faced by the physicians working in these countries. Most of these challenges are sociocultural in origin and a thorough knowledge of the Islamic principles can help overcome these challenges.

We thank Larijani and Zahedi for their detailed review [1] of our article on Patient-Centered Care (PCC) in South Asia [2]. We agree with the authors that the content of the article is not generalizable to the entire Islamic world. Various Islamic countries, spread across the globe, have different socioeconomic and cultural environments, which impact the behavior of their respective communities. Therefore, generalizing any analysis, based on a few countries, to the entire Islamic community, was neither our aim, nor is it wise. We restricted our analysis to South Asia, using India and Pakistan as specific examples. Even though our analysis was based on the Islamic society of India and Pakistan, we included all common barriers to PCC in this region, be they religious or sociocultural. We did not aim to thin the distinction between these two different entities. Culture and religion, as pointed out by the authors [1], are two separate aspects of life. Both of these contribute to the health behaviors of a society and inform the biopsychosocial model of healthcare.

We believe that the analysis of Larijani and Zahedi represents a slight misinterpretation of our statements rather than a conflict of views. The authors mention that the myths of “disease process is related to supernatural entities” or “fighting against disease is in opposition to God’s will” are not acceptable to patients in Iran; which is admirable. A possible explanation for this difference in behavior may be the literacy level in Iran (85%), which is significantly higher than the rate of 63% in India and 55% in Pakistan [3]. We agree that these myths are not related to the Islamic teachings. We hypothesize that the existence of such myths in South Asia may be due to the lack of education, and pre-Islamic sociocultural scenario of the region. That being said, as we mention in the article, awareness and literacy are gradually increasing in South Asia, the patients are more empowered and keener to take part in their own management and take a more pro-active role when it comes to their health. One hopes that South Asia, and other Islamic countries, can improve their literacy level to the extent that Iran has achieved.

The lower literacy level of South Asia may be in concordance with the suboptimal understanding of modern medical knowledge among some traditional religious leaders. Through our article, we aim to sensitize health care professionals to encourage a pro-health partnership with religious leaders, who hold immense influence on the population. We agree that the religious scholars of the day are definitely more informed regarding the latest medical practices, their pros and cons, in comparison with the previous scholars, and have proven their utility in enhancing diabetes care in the region [4].

The authors also point out that the issues raised under the heading of “common myths and challenges in the Islamic society” are related to the cultural traditions of the population. Our intention was not to label these myths as originating from the Islamic teachings but rather highlight them as the projection of sociocultural norms of the Islamic society of South Asia. The authors rightfully state that these myths do not have any basis in the Islamic teachings. We could not agree more.

Islam believes in modernity and encourages patients to take a more pro-active role in their health. Islam promotes the use of PCC and Muslim patients are increasingly moving towards making informed healthcare decisions in partnership with their healthcare professionals. To highlight that Islam supports a pro-active role in healthcare and ensure that the sociocultural issues prevalent in the Islamic society of South Asia are not confused with Islamic teachings, we quoted several Hadiths and verses that clearly reveal that Islam supports modern medicine and is in support of PCC [2].

Nevertheless the importance of culture-bound myths is great, and these need to be handled tactfully and efficiently in order to promote PCC in all societies. It is imperative for all health care professionals to to take a community centered approach to this issue, and to incorporate patient centered care in the daily practice of endocrinology. We thank the journal for highlighting an important aspect of diabetes that is often overlooked.

## Abbreviations

PCC: Patient centered care.
